# Serum and urine metabolomic profiling in Miniature Schnauzer dogs with and without calcium oxalate urolithiasis

**DOI:** 10.1007/s11306-026-02429-1

**Published:** 2026-04-10

**Authors:** Emily L. Coffey, Andres Gomez, Nicole M. Tate, Lauren A. Baker, Jody P. Lulich, Eva Furrow

**Affiliations:** 1 Department of Veterinary Clinical Sciences, University of Minnesota, Saint Paul, MN USA; 2 Department of Animal Science, University of Minnesota, Saint Paul, MN USA; 3 Department of Veterinary Population Medicine, University of Minnesota, Saint Paul, USA; 4 Department of Animal and Dairy Sciences, University of Wisconsin, Madison, WI USA

**Keywords:** Urolithiasis, Urinary stone disease, Dyslipidemia, Calcium oxalate, Dog

## Abstract

**Introduction:**

Calcium oxalate (CaOx) urolithiasis is associated with metabolic disorders, including dyslipidemia. Improved understanding of underlying metabolic derangements is needed. The Miniature Schnauzer presents an opportunity to investigate connections between hyperlipidemia and CaOx stones, as both are prevalent in the breed.

**Objectives:**

To characterize lipidomic (serum) and metabolomic (serum and urine) profiles in Miniature Schnauzers with (cases) and without (controls) CaOx urolithiasis.

**Methods:**

Ultrahigh performance liquid chromatography-tandem mass spectroscopy was performed on serum from cases (*n* = 15) and controls (*n* = 27) for lipidomic and metabolomic analysis. Urine metabolomics was included for a subset of dogs. Ten metabolites with previously established biological links to urolithiasis were prespecified as “high priority.” Cases and controls were compared to identify differentially abundant metabolites (FDR-adjusted q-values).

**Results:**

No lipid species were differentially abundant. Three serum metabolites differed between groups (all lower in cases): 10-undecenoate, N-delta-acetylornithine, and glutarate (q-values 0.005, 0.03, and 0.009, respectively). Cluster analysis of high priority metabolites identified a subset of cases with distinct profiles, characterized by lower citrate and higher phosphate, glycine, and hippurate. Urinary profiles exhibited 202 differentially abundant metabolites, including higher acetylcarnitine and carnitine in cases (q-values 0.002 for both).

**Conclusions:**

No differences in lipids were identified between Miniature Schnauzers with and without CaOx stones. Distinct metabolic subsets of stone formers might exist within the breed. Reduced N-delta-acetylornithine in stone formers is also reported in human stone formers and might reflect dietary acid load. Acetylcarnitine and carnitine enrichment in the urine of stone formers also warrants further exploration.

**Supplementary Information:**

The online version contains supplementary material available at 10.1007/s11306-026-02429-1.

## Introduction

Kidney stones are associated with metabolic disturbances such as hypertension, obesity, diabetes mellitus, and dyslipidemia in humans (Cappuccio et al. 1999, 1990; Siener et al. 2004; Taylor et al. 2005aa, 2005b; Torricelli et al. 2014). Calcium oxalate (CaOx) is the most common stone composition (Singh et al. 2015), and stone formers often have differences in the urinary, fecal, and serum metabolome as compared to stone-free controls, particularly related to amino acid and lipid metabolism (Agudelo et al. 2022; Denburg et al. 2020; Duan et al. 2020; Gao et al. 2022; Thongprayoon et al. 2022; Wang et al. 2019; Wen et al. 2021). Altered lipid profiles have been associated with CaOx stone risk, though findings are inconsistent across studies (Besiroglu and Ozbek 2019; Kang et al. 2014; Sur et al. 2013; Torricelli et al. 2014). Similarly, rodent models of stone formation have alterations in polyunsaturated fatty acids, ceramides, and lysophosphocholines (Chao et al. 2018). The growing evidence that lipid and other metabolic derangements contribute to CaOx stone formation demands further exploration.

Companion dogs frequently form CaOx uroliths and offer a unique opportunity to investigate metabolic pathways involved in stone formation (Alford et al., 2020; Hunprasit et al. 2019). The Miniature Schnauzer is ideal for this area of research as the breed is strongly over-represented for CaOx urolithiasis and has a high prevalence of hypertriglyceridemia (HTG). A previous study identified an association between HTG and CaOx urolithiasis in dogs (Paulin et al. 2022). However, Miniature Schnauzers were only present in the CaOx case group and were absent from the control group. This might have resulted in a spurious association from breed rather than from a true link in the diseases. Investigations within the Miniature Schnauzer breed have the potential to clarify the link between HTG and CaOx uroliths and ascertain specific lipid species or other metabolites involved.

The primary objective of this study was to identify differences in serum lipidomic and untargeted metabolomic profiles between Miniature Schnauzers with and without CaOx urolithiasis. A secondary objective was to identify differences in untargeted urine metabolomic profiles between Miniature Schnauzers with and without CaOx urolithiasis. We hypothesized that CaOx stone formers exhibit distinct lipidomic and metabolomic signatures as compared to stone-free controls.

## Methods

### Sample selection

Serum samples were selected from a biobank at the University of Minnesota Canine Genetics Laboratory from studies on HTG and CaOx urolithiasis in Miniature Schnauzers (Furrow et al. 2016, 2015; Smith et al. 2017). For a subset of dogs, urine was available from the same visit. All samples were stored at−80˚ C until analysis; any freeze-thaw cycles were recorded. Cases included Miniature Schnauzers with a history of confirmed CaOx urolithiasis, defined as ≥ 70% CaOx composition as diagnosed by polarized light microscopy and infrared spectroscopy. Controls were Miniature Schnauzers at least 8 years of age (the average age for stone formation) without a diagnosis of urolithiasis and no uroliths observed on abdominal radiographs (Hunprasit et al. 2019).

Dogs were excluded if they had chronic kidney disease, an endocrinopathy (diabetes mellitus, hypercortisolism, or hypothyroidism), or hypercalcemia diagnosed prior to or within one year of sample collection. Fasting serum triglyceride (TG) concentrations were required for inclusion, and HTG was defined as TG concentrations exceeding 108 mg/dL (Tate et al. 2024; Xenoulis et al. 2007). Diagnostic results, medical interventions, diet, and co-morbidities were extracted from medical records. This included concentrations of blood urea nitrogen (BUN), creatinine, total and ionized calcium levels, glucose from serum or blood chemistry profiles, and results of spot urine calcium-to-creatinine ratios (UCa: Cr) if available.

### Sample and data processing

Frozen serum and urine samples were placed on dry ice prior to shipping to Metabolon, Inc. (Durham, NC). Upon arrival, samples were maintained at −80˚ C until processed for the Complex Lipid Panel (serum only; up to 1,100 lipid species) and Global Metabolomics HD4 (serum and urine; up to 5,400 metabolites) using the automated MicroLab STAR^®^system (Hamilton Company, Reno, NV). Ultrahigh performance liquid chromatography-tandem mass spectroscopy (UPLC-MS/MS) was performed with raw data extraction and compound identification. Area-under-the-curve analysis was used for metabolite peak quantification of raw data. Sample processing, internal standards and quality control measures, chromatographic alignment, and compound identification were completed in accordance with Metabolon’s standard protocols (Evans et al. 2014; Ford et al. 2020).

Data processing and statistical analyses were performed using MetaboAnalyst 6.0 (Pang et al. 2024) statistical software (v. 4.0.2). Features with more than 50% missing values or with near-constant or single values across all samples were removed prior to downstream analysis using the interquartile range. For the remaining compounds, missing values were imputed with half the minimum value (Wei et al. 2018). Serum data normalization was performed using row-wise normalization by sum and generalized log transformation, and data were Pareto-scaled via mean-centering and dividing by the square root of the standard deviation of each variable. Urinary data were normalized by urine osmolality, instrument batch, and log transformation.

Normality of clinical data were determined using the Shapiro-Wilks test. Student’s *t* tests were used to compare normally distributed continuous clinical variables between cases and controls: age and serum/blood concentrations of creatinine, BUN, ionized calcium, total calcium, and glucose. Wilcoxon rank-sum tests were used to compare ordinal data or data lacking a normal distribution: body condition score (BCS), UCa: Cr, serum TGs, and freeze-thaw cycles. Fisher’s exact tests were used to compare proportions of sex and patients with HTG between groups.

Log fold change of case to control groups was calculated for each lipidomic and metabolomic feature, and Student’s *t* tests were used to compare group means. Correction for multiple comparisons was performed using the Benjamini-Hochberg procedure, and statistical significance for differential abundance was defined as a false discovery rate (FDR)-adjusted *P* value (q-value) < 0.10. Metabolites identified as significant in the primary analysis were further evaluated in multivariable linear models to assess whether associations persisted after adjusting for sex. Clustering of cases and controls was visualized with principal component analysis (PCA). A permutational analysis of variance (PERMANOVA) with 1,000 permutations was performed to determine statistical differences using Euclidean distances between groups (*adonis2* function), and multivariable PERMANOVA was performed to assess independent and interactive effects of stone status and HTG status. Partial least squares discriminant analysis (PLS-DA) models were also examined. Features with the highest discriminatory power between case and control groups were identified with random forest analysis.

Prior to analysis, a subset of 10 metabolites with known biological links to CaOx stone formation were labelled as “high priority” (Table S1). Correlation analyses between high priority metabolites were performed using Pearson’s correlations. PCA was performed for the subset, and the Hopkins statistic was used to evaluate their clustering tendency with ≥ 0.5 considered non-random or clusterable. The ‘fviz_nbclust’ package in R was used to select the optimal number of clusters, and clustering analysis of the high priority metabolites was performed using Euclidean distance measures and the ward.D clustering algorithm.

## Results

### Study population

Out of 95 biobanked serum samples from Miniature Schnauzers, 47 met the criteria for a CaOx case or control. Of those, four were excluded for a diagnosis of an endocrinopathy and one for chronic kidney disease. This left 42 dogs, comprising 15 cases and 27 controls. Five samples (1 case and 4 controls) were only analyzed with the Complex Lipid Panel due to small volumes (< 100 µL). Paired urine samples collected on the same day as serum were available for 18 dogs (8 cases and 10 controls). One dog was an outlier across the serum analyses, driven by theobromine levels over 100 times the average value of other serum samples. Rather than exclude this dog, theobromine was removed from the metabolite dataset prior to downstream analysis. After removal of theobromine, the dog was no longer an outlier.

Patient metadata for the 42 study dogs are summarized in Table 1. Males comprised most cases (13/15, 87%) compared to approximately half of the controls (14/27, 52%; *P* = 0.042). There was a small difference in serum/blood glucose concentrations between groups, with the case average 10 mg/dL greater than the control average (*P* = 0.026). Averages for both groups were within the normal reference ranges. The other variables tested did not differ significantly between groups. Of the 15 cases, 10 were recurrent stone formers, and the mean age of first stone diagnosis was 7.5 years (± 2.6). Eleven of the cases had stones documented at the time of sample collection (i.e., active cases). Three dogs did not have active stones, with the last stone episode within one month for two dogs and five years for the third dog. Stone status at the time of sampling was unknown for the remaining dog. Eleven dogs had lower urinary tract (bladder or urethral) stones, and three had both lower urinary tract and kidney stones. One dog had only kidney stones at the time of enrollment, but two years later developed cystoliths that were removed and confirmed to be CaOx. Four of the cases were being fed a therapeutic stone prevention diet (*n* = 2, Royal Canin Urinary SO dry dog food; *n* = 1, Royal Canin Urinary SO Moderate Calorie dry dog food, Royal Canine, St. Charles, MO; *n* = 1, Hill’s Prescription Diet u/d dry dog food, Hill’s Pet Nutrition, Inc., Topeka, KS), though all but one received non-therapeutic treats. Controls consumed a variety of commercial diets, but none were fed therapeutic stone prevention diets. Medications are summarized in Table S2.

**Table 1:** Summary of metadata for 42 serum samples from Miniature Schnauzers with (*n* = 15) and without (*n* = 27) CaOx urolithiasis, reported as mean ± standard deviation or median (range)

Variable	Cases (*n* = 15)	Controls (*n* = 27)	*P* value
Age (years)	9.9 ± 2.2	10.3 ± 1.4	0.62
Sex	2FS, 13MN	13FS, 14MN	0.042
BCS (1–9 scale)	6 (4–8)	5 (3–7) [25]	0.22
UCa: Cr (mg/mg)	0.034 (0.011–0.14) [14]	0.023 (0.007 to 0.11)	0.32
TG (mg/dL)	130 (24–973)	70 (14–2089)	0.48
Proportion with HTG	8/15 (0.53)	12/27 (0.44)	0.75
BUN (mg/dL)	12.8 ± 4.9 [14]	14.8 ± 5.6 [25]	0.29
Creatinine (mg/dL)	0.8 ± 0.2 [14]	0.8 ± 0.2 [25]	0.89
Glucose (mg/dL)	110 ± 13	100 ± 12 [25]	0.026
Ionized calcium (mg/dL)	5.4 ± 0.24	5.4 ± 0.20 [16]	0.85
Total calcium (mg/dL)	10.3 ± 0.7 [5]	10.0 ± 0.6 [7]	0.52
Freeze-thaw cycles	2 (1–3)	2 (1–3)	0.62

The number of samples measured is reported in brackets when not assessed in all dogs. *BCS* body condition score, *BUN* blood urea nitrogen, *FS* female spayed, *HTG* hypertriglyceridemia, *MN* male neutered, *TG* triglycerides, *UCa Cr* urine calcium-to-creatinine ratio

### Serum lipidomics

A total of 988 unique lipid metabolites were identified from the 42 serum samples. After data filtering, 956 metabolites from 14 lipid classes remained for downstream analysis.

**Fig. 1:**
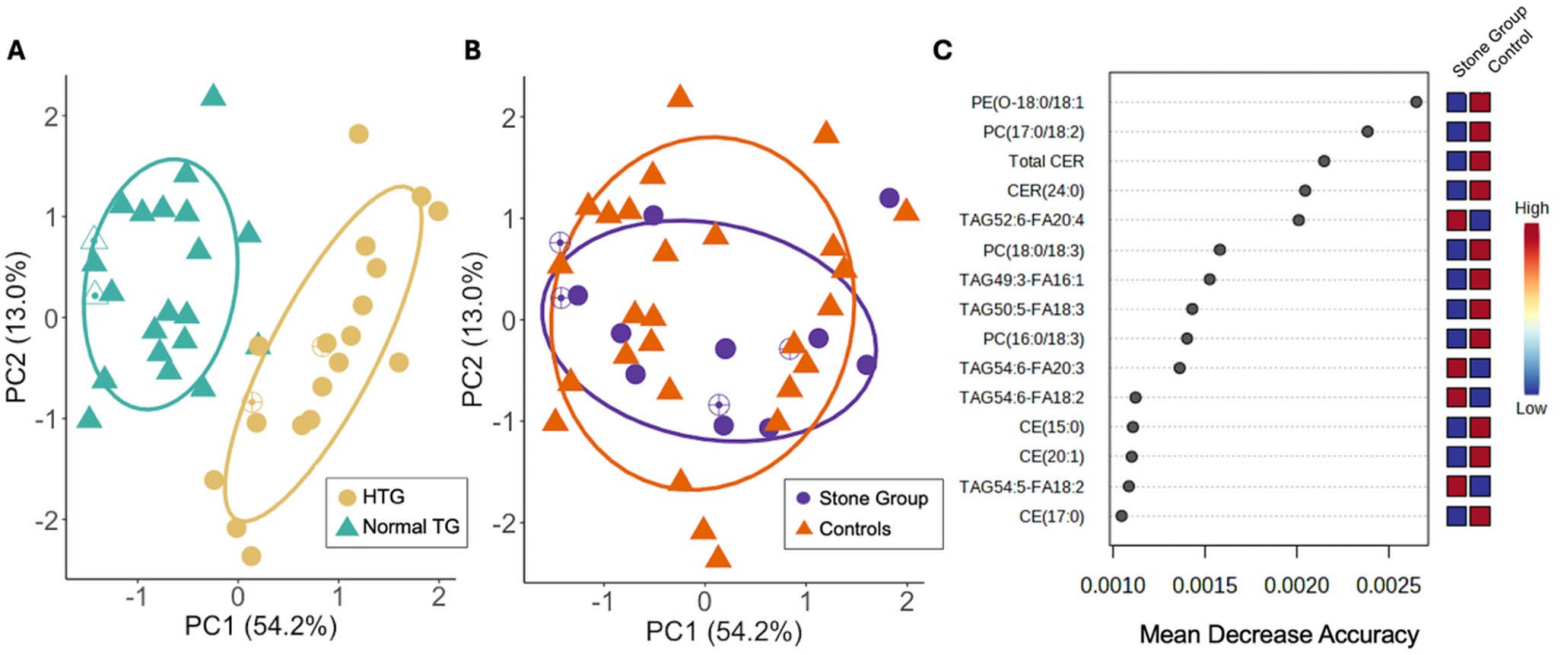
Lipidomic profiles in Miniature Schnauzers with calcium oxalate (CaOx) urolithiasis (*n* = 15) and stone-free controls (*n* = 27), with or without hypertriglyceridemia (HTG). Principal component analysis (PCA) of serum lipidomic data from Miniature Schnauzers with **A** HTG (yellow circles) and normal triglyceride (TG) levels (teal triangles; *P* = 0.001, R^2^ = 0.39), and **B** CaOx stone cases (purple circles) and stone-free controls (orange triangles; *P* = 0.36, R^2^ = 0.015). Open triangles and open circles represent dogs consuming a therapeutic urinary stone prevention diet. Ellipses represent the 95% confidence interval for each group. Percent variation of each principal component is included in parentheses. **C** Fifteen lipid metabolites identified by random forest analyses as having the most discriminatory power between CaOx stone cases and stone-free controls

Sixteen features had a *P*_raw_ value < 0.05 in the differential abundance analysis (Table S3); none remained significant after adjustment for multiple comparisons. A PCA plot of lipidomic features showed distinct clustering by HTG status but failed to demonstrate clear clustering by stone status (Fig. 1A and B). In multivariable PERMANOVA, HTG remained strongly associated with lipidomic composition (*P* = 0.001, R^2^ = 0.39), whereas neither stone status nor the interaction between stone status and HTG were significant (*P* = 0.36, R^2^ = 0.015, and *P =* 0.70, respectively). PLS-DA models showed poor predictive performance by stone status, with Q2 values below zero across all tested components. Random forest analysis had low accuracy for stone status prediction; 0/15 cases and 21/27 controls were accurately classified with an out-of-bag error of 0.5. The lipid features with the highest discriminatory power all had mean decrease in accuracy values < 0.3% (Fig. 1C).

### Serum untargeted metabolomics

**Fig. 2:**
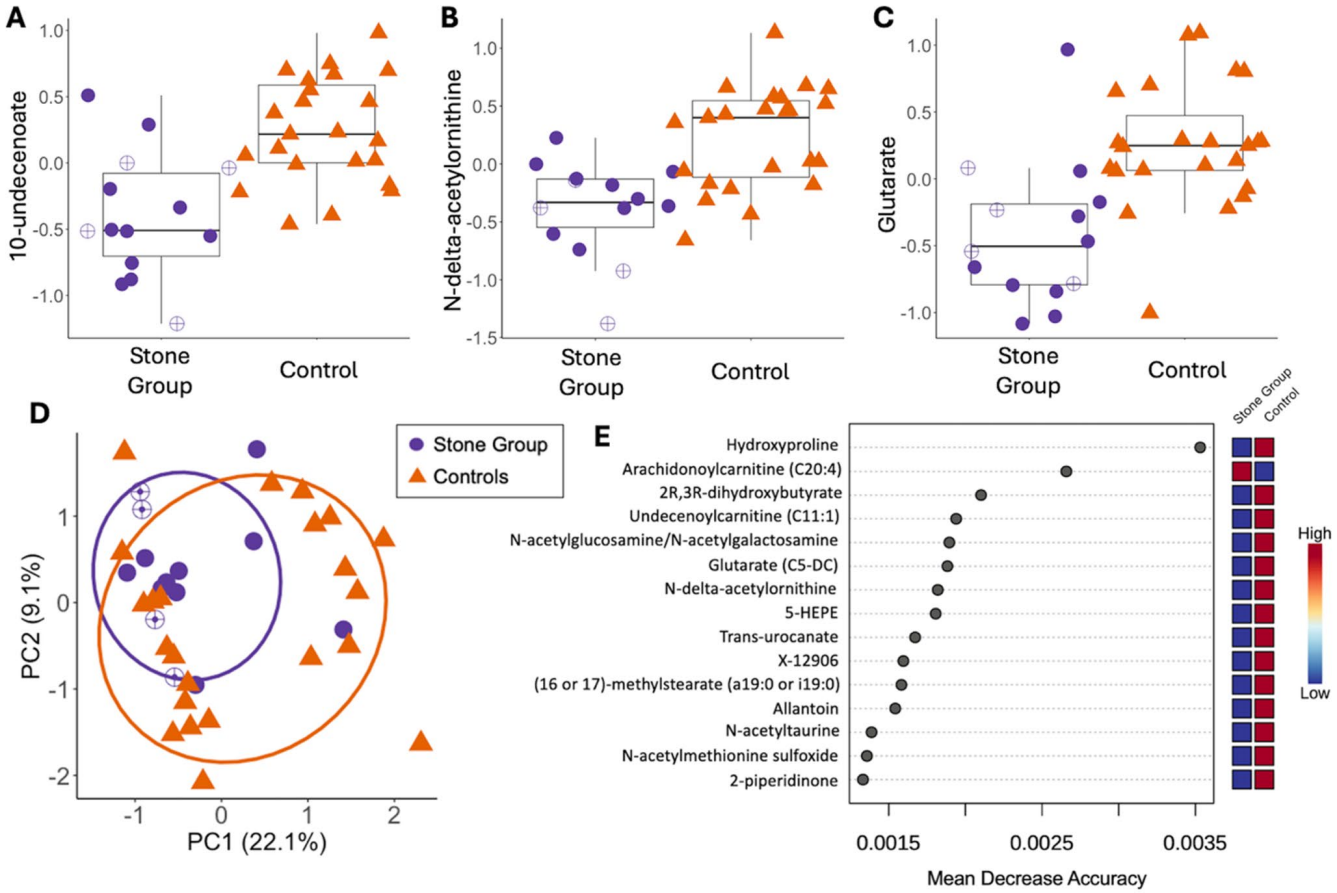
Untargeted serum metabolomic profiles between Miniature Schnauzers with CaOx urolithiasis (*n* = 14, purple circles) and stone-free controls (*n* = 23, orange triangles). Open circles represent dogs consuming a therapeutic urinary stone prevention diet. Boxplots of differentially abundant metabolites between groups, including normalized levels of **A** 10-undecenoate (*P* = 0.002; q-value = 0.005), **B** N-delta-acetylornithine (*P =* 0.03; q-value = 0.03), and **C** glutarate (*P* = 0.006; q-value = 0.009). The boxes represent the 25th and 75th percentiles, the horizontal lines represent the median, and the whiskers represent 1.5 times the interquartile range. **D** Principal component analysis (PCA) of serum metabolite data from cases and stone-free controls (*P* = 0.023, R^2^ = 0.05). Ellipses represent the 95% confidence interval for each group. Percent variation of each principal component is included in parentheses. **E** Fifteen metabolites identified by random forest analyses as having the most discriminatory power between cases and controls

A total of 802 unique metabolites were identified from the 37 serum samples. After data filtering, 569 metabolites remained for downstream analysis. Fifty-nine were unnamed, and 510 metabolites had a known identity. Eighty-four features had a *P*_raw_ value < 0.05 for the differential abundance analysis (Table S4), and three had a q-value < 0.10: 10-undecenoate, N-delta-acetylornithine, and glutarate (Fig. 2A-C). After adjusting for sex, each metabolite had a q-value < 0.05.

PCA plots of untargeted metabolomics demonstrated partial clustering by stone status (Fig. 2D; PERMANOVA *P* = 0.023, R^2^ = 0.05). PLS-DA models showed poor predictive performance, with Q2 values < 0.1 across all tested components. Random forest analysis had low accuracy for stone status prediction; 6/14 cases and 21/23 controls were accurately classified with an out-of-bag error of 0.27. The metabolites with the highest discriminatory power between cases and controls had mean decrease in accuracy values < 0.4% (Fig. 2E). Hydroxyproline was identified as the metabolite with the most discriminatory power with increased abundance observed in the serum of control dogs. No major differences were observed in global serum metabolomic profiles between dogs with (*n* = 17) or without (*n* = 20) HTG (PERMANOVA *P* = 0.26, R^2^ = 0.03).

### Targeted analysis of high priority serum metabolites

**Fig. 3:**
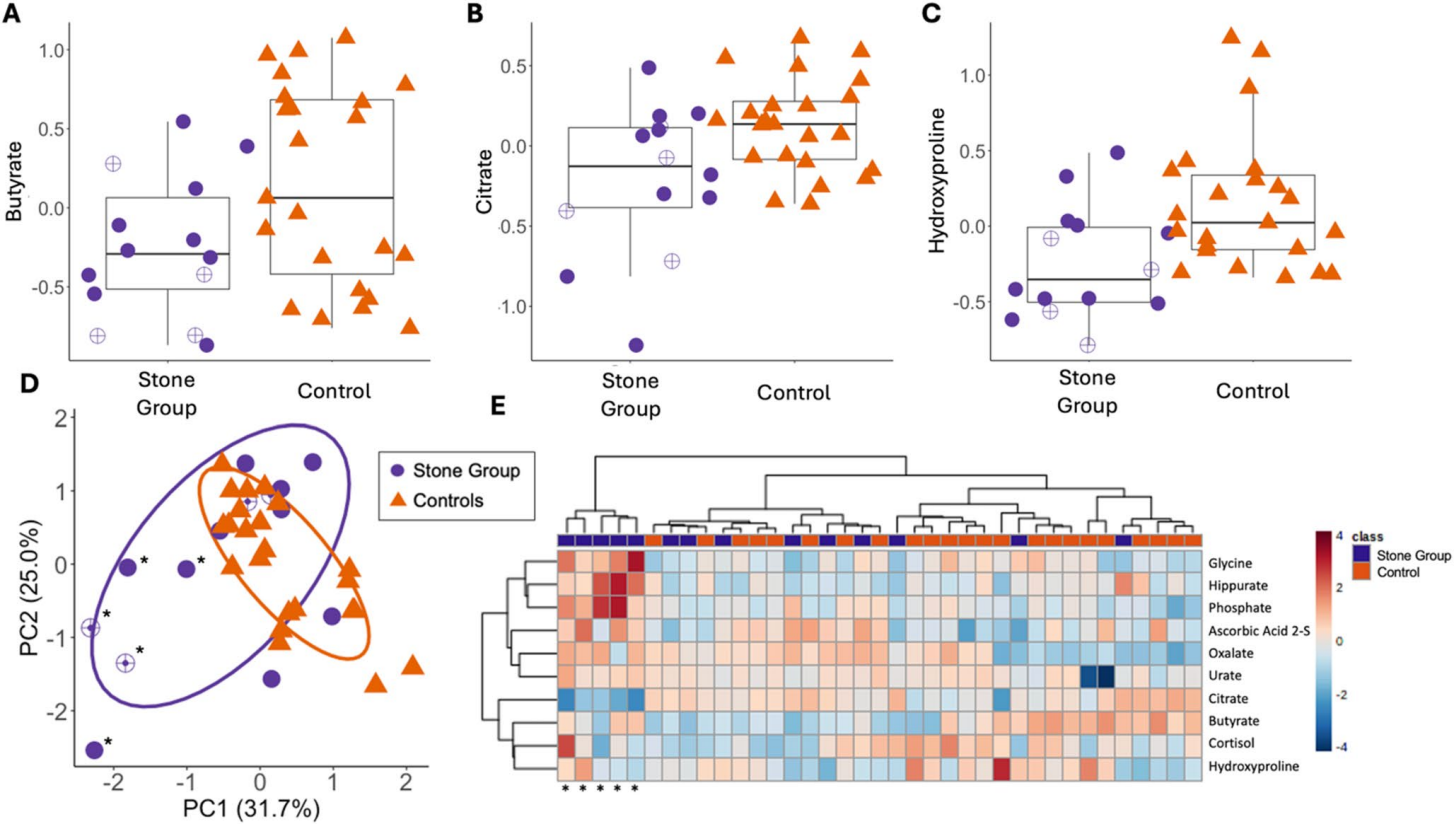
Profiles of 10 high priority metabolites between Miniature Schnauzers with CaOx urolithiasis (*n* = 14, purple circles) and stone-free controls (*n* = 23, orange triangles). Open circles represent dogs consuming a therapeutic urinary stone prevention diet. Boxplots of differentially abundant metabolites, including normalized levels of **A** butyrate (*P* = 0.049; q-value = 0.33), **B** citrate (*P* = 0.011; q-value = 0.19), and **C** hydroxyproline (*P* < 0.01; q-value = 0.19). The boxes represent the 25th and 75th percentiles, and the whiskers represent 1.5 times the interquartile range. **D** Principal component analysis (PCA) of high priority metabolite data from cases and stone-free controls (*P* = 0.029, R^2^ = 0.07). Ellipses represent the 95% confidence interval for each group. Percent variation of each principal coordinate is included in parentheses. **E** Heatmap of the high priority metabolites based on a dendrogram from clustering analysis. Each column represents a single sample, with cases identified in purple and controls in orange. The 5 cases with distinct clustering based on the targeted panel are signified by an asterisk (*) in panels **D** and **E**

Table 2 summarizes the differential abundance analysis results for each of the high priority metabolites. Three high priority metabolites (butyrate, citrate, and hydroxyproline) were lower in cases and had *P*_raw_ < 0.05, though none met the FDR threshold when corrected across the full dataset Fig. 3A-C).

The PCA plot of the high priority metabolites showed overlap in groups, with stone-free control dogs exhibiting tighter clusters compared to cases (Fig. 3D; PERMANOVA *P* = 0.029, R^2^ = 0.07). Hierarchical clustering with a heatmap is displayed in Fig. 3E. The Hopkins statistic was 0.64, and the ideal number of clusters was determined to be 4. Five cases separated from all other dogs at the first dendrogram branch. These dogs exhibited a distinct profile characterized by lower citrate and higher glycine, hippurate, and phosphate. All five dogs were male, three had recurrent stone disease (four confirmed active cases), and two were fed therapeutic stone prevention diets.

There were strong negative correlations between serum glycine and citrate (*P* < 0.0001; rho = −0.81), serum butyrate and oxalate (*P* < 0.0001; rho = −0.63), and serum phosphate and citrate (*P* < 0.0001; rho = −0.68).

**Table 2:** Targeted panel of ten metabolites selected for biological relevance to CaOx urolith formation in Miniature Schnauzers with and without CaOx urolithiasis

Metabolite	Log2 fold change (Case vs. Control)	Student’s t test Raw *P* value, (FDR q-value)
Ascorbic acid 2-sulfate		
Serum	−0.23	0.17, (0.49)
Urine	1.81	0.005, (0.05)
Butyrate		
Serum	−1.00	0.049, (0.33)
Urine	0.22	0.80, (0.88)
Citrate		
Serum	−0.38	0.011, (0.19)
Urine	0.38	0.40, (0.57)
Cortisol*		
Serum	−0.48	0.053, (0.34)
Urine	NA	NA
Glycine		
Serum	−0.047	0.57, (0.77)
Urine	2.76	0.002, (0.032)
Hippurate		
Serum	2.33	0.21, (0.51)
Urine	1.23	0.25, (0.43)
Hydroxyproline		
Serum	−0.68	0.0099, (0.19)
Urine	0.087	0.26, (0.43)
Oxalate		
Serum	0.36	0.27, (0.54)
Urine	1.70	0.10, (0.26)
Phosphate		
Serum	0.76	0.092, (0.40)
Urine	1.65	0.007, (0.063)
Urate		
Serum	−0.11	0.58, (0.77)
Urine	1.19	0.18, (0.35)

*Not available in urine untargeted metabolomic data

### Urinary metabolomics

Urine samples were available from 8 cases and 10 control dogs. All 8 cases were male, whereas only 3 of the control dogs were male (*P* = 0.004, Figure S1). BUN values were higher in control dogs (*P* = 0.01), but values from both groups remained within the normal reference range. Other variables were similar between groups. Additional metadata are presented in Table S5. A total of 1,195 unique metabolites were identified from the 18 urine samples. After data filtering, 1,189 metabolites remained for downstream analysis (369 unnamed, 820 with known identity).

**Fig. 4:**
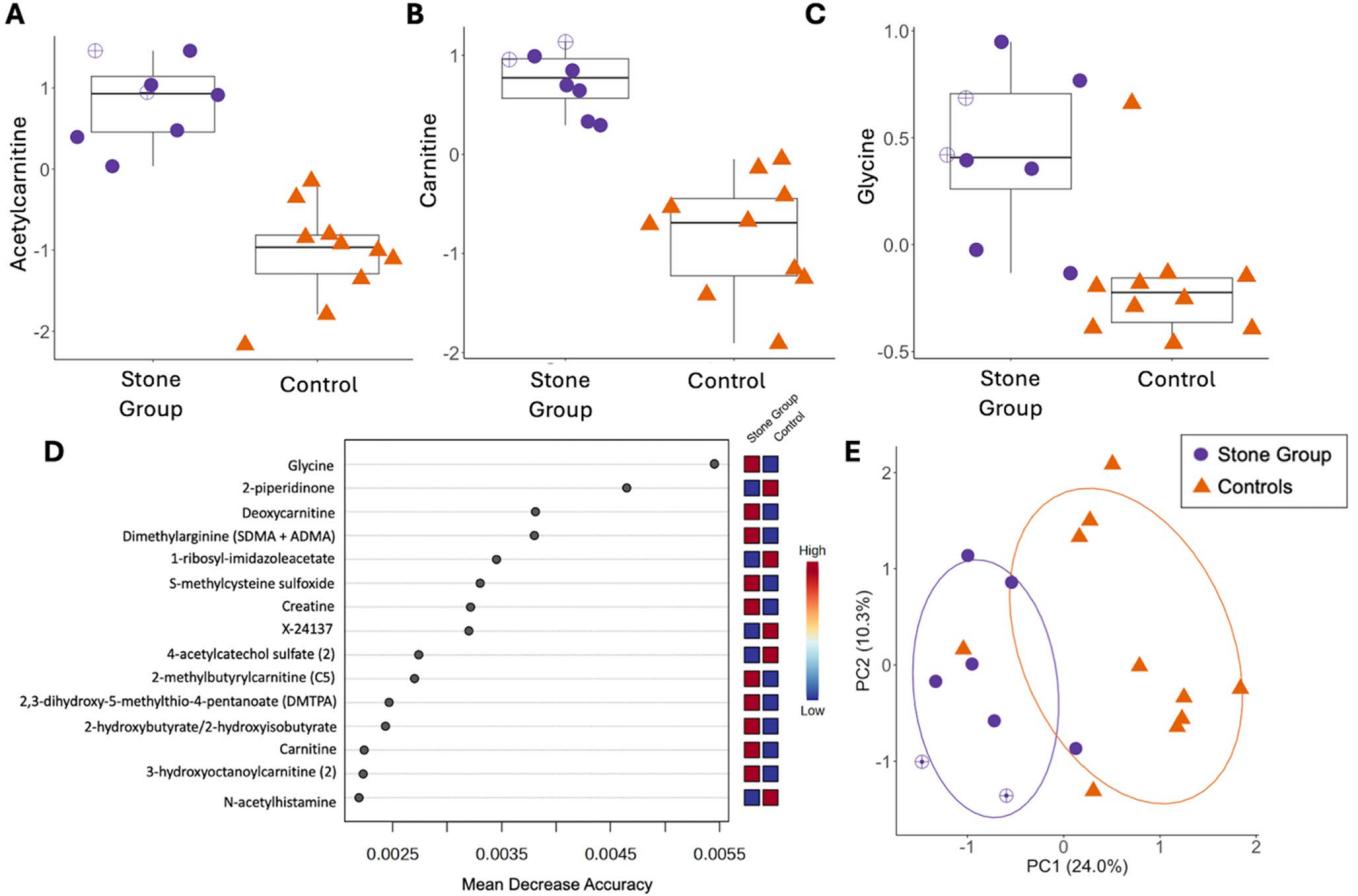
Untargeted urine metabolomic profiles between Miniature Schnauzers with CaOx urinary stones (*n* = 14, purple circles) and stone-free controls (*n* = 23, orange triangles). Open circles represent dogs consuming a therapeutic urinary stone prevention diet. Boxplots of differentially abundant metabolites between groups, including normalized levels of **A** acetylcarnitine (*P* < 0.0001; q-value = 0.002) and **B** carnitine (*P* < 0.0001; q-value = 0.002). The boxes represent the 25th and 75th percentiles, the horizontal lines represent the median, and the whiskers represent 1.5 times the interquartile range. **C** Boxplot of normalized levels of glycine (*P* < 0.01; q-value = 0.032). **D** Fifteen metabolites identified by random forest analyses as having the most discriminatory power between cases and controls. **E** Principal component analysis (PCA) of serum metabolite data from cases and stone-free controls (*P* = 0.003, R^2^ = 0.17). Ellipses represent the 95% confidence interval for each group. Percent variation of each principal coordinate is included in parentheses

Differential abundance analysis discovered 334 features with a *P*_raw_ value < 0.05, and 202 had a q-value < 0.10 (Table S6). Of the 202 differentially abundant metabolites, 107 (53%) had greater abundance in cases. Acetylcarnitine and carnitine were the most differentially abundant named metabolites and were both higher in cases (q-value = 0.002 for both; Fig. 4A-B). An unnamed differentially abundant metabolite was identified as X-21842 and was higher in controls (q-value = 0.002).

Random forest analysis had high accuracy for stone status prediction; 7/8 cases and 9/10 controls were accurately classified with an out-of-bag error of 0.11. Glycine was higher in cases and had the highest discriminatory power (Fig. 4C-D). The metabolites with the highest discriminatory power had mean decrease in accuracy values < 0.6% (Fig. 4D). PCA plots of untargeted urine metabolomics demonstrated distinct clustering by stone status (Fig. 4E; PERMANOVA *P* = 0.003, R^2^ = 0.17), and PLS-DA models demonstrated good predictive performance (Q2 > 0.5). Half of the cases (*n* = 4) and controls (*n* = 5) with urine analyses performed were classified as HTG. Urine metabolomic profiles also differed by HTG status (PERMANOVA *P* = 0.02, R^2^ = 0.11).

Nine of the high priority serum metabolites were also measured in urine samples (Table 2). Of these, glycine, ascorbic acid 2-sulfate, and phosphate were higher in cases (q-value < 0.10). There were strong correlations between urine glycine and ascorbic acid 2-sulfate (*P* < 0.001; rho = 0.75), urine butyrate and oxalate (*P* < 0.01; rho = 0.69), and urine glycine and urate (*P* < 0.01; rho = 0.65). Serum butyrate was negatively correlated with urine oxalate (*P* = 0.039; rho = −0.49).

## Discussion

We did not detect differences in fasting TG concentrations or lipidomic profiles between CaOx cases and controls. This suggests that dyslipidemia is unlikely a major risk factor for CaOx urolithiasis in Miniature Schnauzers. Serum metabolomic analysis identified three metabolites with differential abundance between CaOx cases and controls. When prespecified high priority metabolites were analyzed with clustering, five CaOx cases had a divergent profile from the other dogs; this might indicate a unique metabolic subset of stone formers. Urine metabolomics showed over 200 differentially abundant metabolites by stone status with enrichment of acetylcarnitine, carnitine, and glycine in stone formers.

The absence of statistically significant differences in fasting serum TG concentrations and lipid species between groups aligns with existing human data. In human stone formers, HTG is primarily associated with urinary uric acid excretion and uric acid stone risk rather than CaOx stone risk (Torricelli et al. 2014). In the previous study in dogs that associated higher serum TG concentrations with CaOx stone risk, there was a major breed imbalance with Miniature Schnauzers only present in the case group (Paulin et al. 2022). When Miniature Schnauzers were removed from the analysis and other factors such as age and body condition were considered, the association was no longer present. While lipidomic profiles did not differ by stone status here, there were marked differences according to HTG status, suggesting that it accounted for the majority of observed variation in lipid profiles. Interestingly, urinary metabolomic profiles also differed by HTG status, whereas serum metabolomic profiles were similar between groups. The findings of the current study suggest that HTG and CaOx urolithiasis commonly co-occur in Miniature Schnauzers but do not support a major role of HTG or dyslipidemia in risk for CaOx urolithiasis.

Only three serum metabolites differed between cases and controls: 10-undecenoate, N-delta-acetylornithine, and glutarate. 10-undecenoate is a medium-chain fatty acid, and lower blood levels in humans are associated with a higher Healthy Eating Index (HEI), which is characterized by high consumption of fruits, vegetables, and wholegrains (Noerman and Landberg 2022). The CaOx cases had lower 10-undecenoate relative to controls, which is unexpected, as the HEI is associated with lower risk of kidney stones in humans (Yin et al. 2022). In contrast, N-delta-acetylornithine, an amino acid that was also lower in cases, has a positive correlation with the HEI (Bagheri et al. 2021). N-delta-acetylornithine is also decreased in the plasma of kidney stone formers in the Nurses’ Health Study II cohort (Ferraro et al. 2024) and has an inverse correlation with net endogenous acid production in humans with chronic kidney disease (Rebholz et al. 2019). Glutarate, also lower in the CaOx stone formers, is the conjugate base of glutaric acid and is produced during metabolism of certain amino acids, such as lysine and tryptophan (Zhang et al. 2018). Given potential links between each of these metabolites and dietary components, their differential abundances might reflect differences in diets consumed by the case and control dogs. Differential abundance of these metabolites could also be related to differences in the gut microbiome, as shifts in the microbiome are associated with CaOx stone risk and can impact nutrient metabolism (Coffey et al. 2022; Gnanandarajah et al. 2012). Additionally, as more males were present in the case group, some contribution of sex-related metabolic differences cannot be excluded, although significant metabolites remained associated with disease status after adjustment for sex.

Of the 10 high priority metabolites with known biological relevance to CaOx stone formation, serum hydroxyproline, butyrate, and citrate were lower in cases (*P*_raw_< 0.05), but none achieved the threshold for statistical significance after correction for multiple comparisons. Hydroxyproline is introduced through exogenous (dietary ingestion) and endogenous (collagen turnover) sources, and it contributes to glyoxylate synthesis (Fargue et al. 2018). Glyoxylate is a precursor to oxalate, and increased hydroxyproline can subsequently increase urinary oxalate and CaOx stone risk (Fargue et al. 2018)(Khan et al. 2006; Knight et al. 2006).

Butyrate was not only lower in the serum of dogs with stones, but it also exhibited negative correlations with serum and urinary oxalate levels. Butyrate is a short chain fatty acid (SCFA) that is produced by microbial fermentation of dietary fiber in the gut (Ríos-Covián et al. 2016). In rodent models of CaOx nephrolithiasis, supplementation of butyrate and other SCFAs alters oxalate homeostasis and reduces renal CaOx crystallization (Liu et al. 2021). Additionally, butyrate-producing bacteria and genes related to butyrate production are deficient in the gut microbiome of human CaOx stone formers, as compared to stone-free controls (Choy et al. 2024; Denburg et al. 2020). Collectively, these findings suggest that SCFAs might play protective roles against stone formation.

Citrate has several known anti-lithogenic effects in the urine, including inhibition of CaOx crystallization, chelation of calcium, increasing stone inhibitor activity (e.g., uromodulin), and lowering expression of stone promoters (e.g., osteopontin) (Khan 2018). In addition to being lower in the serum of cases overall, low citrate was also a feature that characterized five CaOx stone formers that clustered separately from the other dogs. Two of these five dogs were consuming prescription urinary diets supplemented with citrate, making the lower values even more notable in these dogs. Hypocitraturia is a common finding in human stone formers, with lower urine citrate levels observed in both clinical urine chemistry panels and in urinary metabolomic studies (Duan et al. 2020; Eisner et al. 2012). While a previous study in Miniature Schnauzers did not identify differences in urinary citrate levels between CaOx stone formers and controls (Lulich et al. 1991), the frequency of hypocitraturia in canine stone formers requires more investigation. In the present study, reduced urinary citrate was not observed in the urine of stone formers relative to stone-free dogs, though only a small number of dogs had paired urine available.

Urinary metabolomic profiles exhibited more pronounced differences between groups than serum, with both acetylcarnitine and carnitine enriched in dogs with stones. These metabolites play roles in fatty acid beta oxidation, and enrichment has also been observed in the urine of human kidney stone patients (Wang et al. 2019). Increases are speculated to occur secondary to kidney epithelial cell damage, which can affect increased transport and excretion of these metabolites into the urine (El-Hattab and Scaglia 2015). However, many stone forming dogs in this population had only bladder stones without detection of concurrent kidney stones, suggesting that factors beyond kidney epithelial damage might contribute to these differences. A key consideration is that 7 of 10 control dogs with available urine samples were female, whereas all dogs in the case group were male. Given the strong association between disease group and sex, combined with the small number of available urine samples, it is difficult to determine whether differences were driven by sex or stone status, representing a key limitation of the urinary metabolomics analysis. Serum and plasma carnitines have been reported to be higher in human males, which is suspected to be driven by hormonal differences (Reuter et al. 2008; Takiyama and Matsumoto 1998). Although all dogs in this study were spayed or neutered, which reduces hormonal variation, a sex-related effect cannot be excluded. Increased carnitine in the urine of dogs with CaOx uroliths has been previously observed, both in comparison to healthy dogs and to dogs with other urolith types (Xu et al. 2024). However, that population similarly had a higher proportion of males in the CaOx stone group relative to controls. The independent association of acetylcarnitine and carnitine with stone formation across multiple species and multiple studies is notable.

Glycine was also higher in the urine of stone formers and was the most discriminatory metabolite on random forest analysis. Glycine is metabolized to the oxalate precursor, glyoxylate, and might decrease oxalate and increase citrate excretion in the urine (Lan et al. 2021). Interestingly, urinary glycine is decreased in human stone formers when compared to stone-free individuals (Duan et al. 2020). Two other high priority metabolites were increased in urine of stone formers: ascorbic acid 2-sulfate and phosphate. Ascorbic acid is the main dietary precursor to endogenous oxalate production (Crivelli et al. 2020; Knight et al. 2016), and increased urinary phosphate loss might contribute to hypercalciuria and CaOx stone risk (Prié et al. 2004).

The small sample size and heterogenous study population are limitations of this study. Random sampling error might have resulted in a different study group than what is seen in the larger population. In fact, the degree of hypercalciuria was less severe in the stone forming Miniature Schnauzers here than what has previously been reported (Carr et al. 2020; Furrow et al. 2015). It is also possible that, although many dogs had stones at the time of serum sampling, the metabolic state that initially drove stone formation had resolved; additionally, a small number of dogs in the case group did not have active stones at the time of sample collection. Together, these factors may have reduced the ability to detect metabolic disturbances associated with stone development. Dogs with diverse diets and medication histories were included, which might have confounded the ability to detect metabolic abnormalities influenced by these factors. Given the potential influence of diet on both lipidomic and metabolomic profiles, as well as on urolith risk, this variability may have contributed to metabolic heterogeneity and reduced the ability to detect disease-associated differences.

Though differential metabolites remained statistically significant after adjustment for sex, imbalance in sex distribution between case and control groups might still have confounded some findings. Future prospective studies with standardized diets and sex-matched cohorts will be important to validate these findings. Furthermore, abdominal radiography was used to screen control dogs for urolithiasis. While radiography is a common method for urolith screening in companion dogs, advanced imaging techniques are considered more sensitive methods of stone detection in humans (Brisbane et al. 2016). If latent stone formers were inadvertently included in the control population, this could reduce the study’s power to detect differences between groups. Another limitation of this study is that some metabolites of interest to CaOx stone formation, such as vitamin D metabolites (Groth et al. 2019; Ketha et al. 2015), were not captured with the techniques used. Lastly, this study was restricted to Miniature Schnauzers due to the increased risk for both dyslipidemia and CaOx urolithiasis within the breed. Studies to validate these findings across additional breeds, including comparative analyses between high-risk breeds and low-risk breeds for CaOx stone formation, would further inform whether the metabolic shifts observed here reflect more generalizable mechanisms of disease.

## Conclusion

This study did not identify evidence for a role of HTG or dyslipidemia in CaOx urolithiasis in Miniature Schnauzers. One of the three serum metabolites lower in cases, N-delta-acetylornithine, is also reduced in plasma of human stone formers and thus is a compelling candidate for future study. The discovery of a subset of stone formers characterized by a unique pattern of metabolites with biological relevance to stone formation also demands further investigation. Urinary metabolomic profiles exhibited more pronounced differences between case and control groups than serum, with notable enrichment observed in acetylcarnitine, carnitine, and glycine in the urine of dogs with CaOx urolithiasis.

## Supplementary Information

Below is the link to the electronic supplementary material.

Supplementary Material 1

Supplementary Material 2

Supplementary Material 3

Supplementary Material 4

Supplementary Material 5

Supplementary Material 6

Supplementary Material 7

## Data Availability

Processed metabolomics data reported in this study are available via Zenodo: https://doi.org/10.5281/zenodo.18121461.

## Abbreviations

BCS: Body condition score
BUN: Blood urea nitrogen
CaOx: Calcium oxalate
FDR: False discovery rate
HEI: Healthy eating index
HTG: Hypertriglyceridemia
PCA: Principal components analysis
PERMANOVA: Permutational analysis of variance
PLS-DA: Partial least squares discriminant analysis
TG: Triglyceride
UCa:Cr: Urine calcium-to-creatinine ratio
USG: Urine specific gravity
