# Supplementary material for: Serum and urine metabolomic profiling in Miniature Schnauzer dogs with and without calcium oxalate urolithiasis
Source: Metabolomics. 2026 Apr 10;22(2):50. doi: 10.1007/s11306-026-02429-1 (PMC13068756; doi:10.1007/s11306-026-02429-1)
Supplement: Supplementary file 1 — Supplementary Material 1 [file 11306_2026_2429_MOESM1_ESM.pdf]

**Supplementary Table 1.** Targeted panel of metabolites and biological relevance to CaOx urolith formation.

| **Metabolite** | **Biological Relevance** |
|---|---|
| Ascorbic acid 2-sulfate | Metabolite of ascorbic acid, the main dietary precursor for endogenous oxalate production (Crivelli et al., 2020; Knight et al., 2016) |
| Butyrate | Reduces oxalate absorption and urinary oxalate excretion in rodent models of CaOx (Liu et al., 2021) |
| Citrate | Inhibitor of CaOx crystallization (A. Khan, 2018) |
| Cortisol | Increases urinary calcium excretion (Arzoz-Fabregas et al., 2013; Ferrari et al., 2002) |
| Glycine | Metabolized to glyoxylate, an oxalate precursor. Potential role in oxalate and citrate excretion (Lan et al., 2021) |
| Hippurate | Regulates CaOx crystallization, serves as a solvent for CaOx crystals (Atanassova & Gutzow, 2013) |
| Hydroxyproline | Metabolized to glyoxylate, an oxalate precursor, and increases urinary oxalate excretion (Fargue et al., 2018; S. R. Khan et al., 2006; Knight et al., 2006) |
| Oxalate | Primary component of CaOx stone composition |
| Phosphate | Essential roles in calcium homeostasis |
| Urate | Increases calcium oxalate crystallization and precipitation (Grover et al., 1992; Moe & Xu, 2018) |
