# Supplementary material for: Serum and urine metabolomic profiling in Miniature Schnauzer dogs with and without calcium oxalate urolithiasis
Source: Metabolomics. 2026 Apr 10;22(2):50. doi: 10.1007/s11306-026-02429-1 (PMC13068756; doi:10.1007/s11306-026-02429-1)
Supplement: Supplementary file 2 — Supplementary Material 2 [file 11306_2026_2429_MOESM2_ESM.pdf]

Supplementary Table 2. Medications and supplements received by Miniature Schnauzers with and without CaOx urolithiasis. Each row represents an individual dog. Brand information is reported when known.

Stone Status	Sex	Medications and Supplements
Case	MN	Potassium citrate*<br>Carprofen<br>Glucosamine supplement
Case	MN	Hydrochlorothiazide<br>Potassium citrate with cranberry
Case	MN	Deracoxib<br>Clavamox *(Zoetis Inc, Parsippany-Troy Hills, NJ)*
Case	MN	Stempet supplement *(Stemtech Corporation, Miramar, FL)*
Case	FS	Cranberry supplement
Case	MN	Gabapentin†<br>Carprofen†
Control	FS	Diethylstilbestrol<br>Glucosamine supplement
Control	MN	Tylosin
Control	MN	Hip & Joint supplement* *(The Missing Link, East Longmeadow, MA)*
Control	FS	Coenzyme Q-10 supplement<br>Welactin fish oil supplement *(Nutramax Laboratories, Lancaster, SC)*<br>Glycoflex 2 joint supplement *(VetriScience Laboratories, Williston, VT)*<br>Vitamin E supplement

* Not given within 24 hours of serum collection.

† Reportedly only administered as needed. Unknown if given within 24 hours of serum collection.

FS – female spayed; MN – male neutered
