# Supplementary material for: Serum and urine metabolomic profiling in Miniature Schnauzer dogs with and without calcium oxalate urolithiasis
Source: Metabolomics. 2026 Apr 10;22(2):50. doi: 10.1007/s11306-026-02429-1 (PMC13068756; doi:10.1007/s11306-026-02429-1)
Supplement: Supplementary file 6 — Supplementary Material 6 [file 11306_2026_2429_MOESM6_ESM.pdf]

Supplementary Table 5. Summary of metadata for 18 urine samples from Miniature Schnauzers with (n = 8) and without (n = 10) CaOx urolithiasis, reported as mean ± standard deviation or median (range).

**Variable**	**Cases (n = 8)**	**Controls (n = 10)**	***P* Value**
Age (years)	9.1 ± 1.3	10.4 ± 1.6	0.07
Sex	8MN	7FS, 3MN	**0.004**
BCS (1-9 scale)	6 (4-8)	5.5 (4-7)	0.71
UCa:Cr (mg/mg)	0.030 (0.011 – 0.09)	0.030 (0.014 to 0.11)	0.59
TG (mg/dL)	122.5 (41-973)	103.5 (37-2089)	0.56
Proportion with HTG	4/8 (0.5)	5/5 (0.5)	1.0
BUN (mg/dL)	9.9 ± 4.0	16.0 ± 5.2	**0.01**
Creatinine (mg/dL)	0.81 ± 0.2	0.87 ± 0.13	0.55
Glucose (mg/dL)	114.6 ± 15	104 ± 13.5	0.14
Ionized calcium (mg/dL)	5.4 ± 0.26 [7]	5.5 ± 0.22 [5]	0.52
Total calcium (mg/dL)	10.8 ± 0.1 [3]	9.75 ± 1.2 [2]	0.43
Freeze-thaw cycles	2 (2-3)	2 (1-3)	0.76

The number of samples measured is reported in brackets when not assessed in all dogs. BCS – body condition score; BUN – blood urea nitrogen; FS – female spayed; HTG – hypertriglyceridemia; MN – male neutered; TG – triglycerides; UCa:Cr – urine calcium-to-creatinine ratio
