# Supplementary material for: Serum and urine metabolomic profiling in Miniature Schnauzer dogs with and without calcium oxalate urolithiasis
Source: Metabolomics. 2026 Apr 10;22(2):50. doi: 10.1007/s11306-026-02429-1 (PMC13068756; doi:10.1007/s11306-026-02429-1)

Figure S1. Principal component analysis (PCA) illustrating untargeted urine metabolomic profiles between male neutered (n = 11, yellow circles) and female spayed (n = 7, teal triangles) Miniature Schnauzers (P = 0.034, R^2 = 0.09). Ellipses represent the 95% confidence interval for each group. Percent variation of each principal component is included in parentheses.

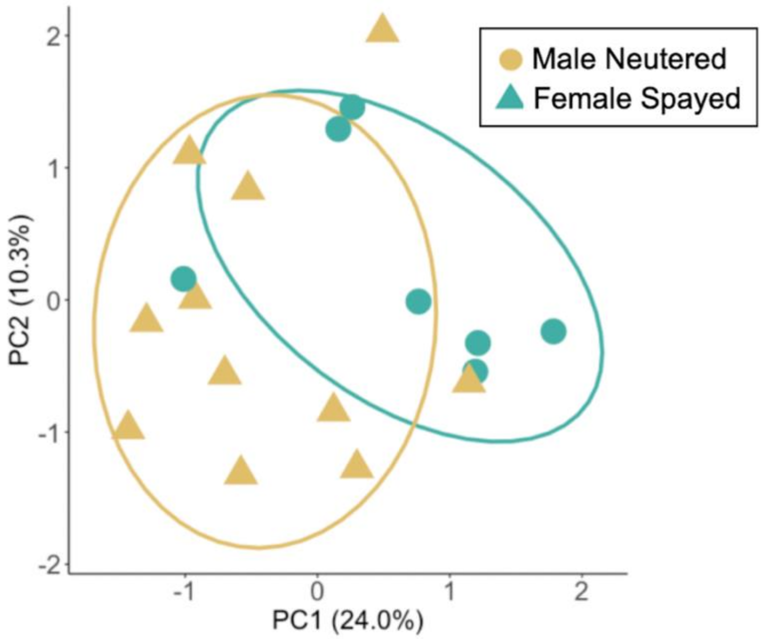

Supplement: Supplementary file 7 — Supplementary Material 7 [file 11306_2026_2429_MOESM7_ESM.pdf]
